# A UK questionnaire survey of current techniques used to perform pelvic organ prolapse repair

**DOI:** 10.1007/s00192-017-3273-z

**Published:** 2017-02-01

**Authors:** Emily Fairclough, Jenny Myers, Anthony Ross Broadhurst Smith, Suzanne Breeman, Fiona Reid

**Affiliations:** 1 The Warrell Unit, Saint Mary’s Hospital, Manchester Academic Health Science Centre, Central Manchester University Hospital NHS Foundation Trust, Oxford Road, Manchester, M13 9WL UK; 2 Division of Development Biology and Medicine, School of Medical Science, Faculty of Biology Medicine & Health, Manchester Academic Health Science Centre, University of Manchester, Manchester, M13 9WL UK; 3 Health Services Research Unit, University of Aberdeen, 3rd Floor, Health Sciences Building, Foresterhill, Aberdeen, AB25 2ZD UK

**Keywords:** Graft, Mesh, Native tissue, Pelvic organ prolapse, Surgical technique

## Abstract

**Introduction and hypothesis:**

Evidence-based medicine should result in better standardisation of practice. This study aims to evaluate whether there remains variation in surgical techniques in native tissue and graft/mesh repairs of pelvic organ prolapse (POP) in UK practice.

**Methods:**

A questionnaire survey was conducted to describe current surgical techniques for native tissue and graft/mesh POP repairs performed by a cohort of UK surgeons recruiting to a large multicentre prolapse trial (PROSPECT).

**Results:**

The questionnaire return rate was 90% (*n* = 56 out of 62). Substantial variations in surgical techniques were seen at every step of the procedure. Native tissue repair: most surgeons used infiltration, 95% (*n* = 53 out of 56), but the volume used varied (10–80 ml). All but one surgeon performed a midline incision; this surgeon performed an elliptical incision. The depth of tissue dissection varied, being both above and below the vaginal muscularis (fascia). Fascial repair methods included midline, closure of separate fascial defects, paravaginal repair and rectal/levator plication. Graft/mesh repairs: many different products and manufacturers were used. There was variation in the method of attachment of graft/mesh inserts and their placement in relation to the fascia. For both native tissue and graft/mesh repairs, the method of fascial dissection, suturing methods and suture material varied. Most surgeons inserted a pack, 91% (*n* = 50 out of 55), soaked in varying substances before use.

**Conclusions:**

There is considerable variation between UK-based surgeons in the surgical techniques used to perform both native tissue and graft/mesh-augmented POP repairs. Further research is required to determine whether these differences influence outcome.

## Introduction

It is frequently quoted that recurrence of pelvic organ prolapse (POP) or incontinence occurs in up to a third of women after surgical repair [1]. In view of discontent with native tissue POP repairs surgeons began to augment repairs with biological grafts or synthetic meshes [2]. In 2010, a national study showed that most UK surgeons continued to perform primary native tissue POP repairs (71%) [3]. However, just over half of the surgeons were performing a graft/mesh repair for secondary POP repairs (56%) [3]. Today, the use of grafts/mesh is more controversial, as there are concerns about the long-term safety of grafts/mesh [4].

Over the last 10 years, questionnaire-based studies have shown that the surgical techniques used to perform native tissue POP repairs and the types of graft/mesh for augmented POP repairs vary between surgeons [5–8]. However, the intraoperative techniques used for the insertion of graft/mesh and whether variations in techniques have any impact on the outcome of the surgery are currently unknown.

The aim of this questionnaire survey was to prospectively describe the current surgical techniques used to perform both native tissue and graft/mesh POP repairs by a cohort of UK surgeons recruiting women to a large multicentre prolapse trial (PROSPECT) [9].

## Materials and methods

This study received ethical approval from the North of Scotland Ethics Committee (NOSRES), (REC/09/SO802/56).

A questionnaire was developed to assess the surgical techniques used by surgeons recruiting to a large, pragmatic, multicentre, randomised controlled trial (RCT) assessing prolapse management (PROSPECT) [9]. The pragmatic nature of the trial gave the opportunity to assess current practices of a cohort of UK surgeons. The questions included were developed in a small focus group of surgeons; then, surgeons from one hospital site checked the face validity. The 52 questions addressed five domains: native tissue techniques for anterior POP repair, native tissue techniques for posterior POP repair, techniques used to perform graft/mesh POP repairs, details on the insertion of packs and the methods used to perform POP-Q (Appendix 1). The study did not consider techniques used to perform vault/apical surgery for prolapse.

In 2010, at the start of the study, the questionnaires were distributed by email to the lead research nurse at each site. The research nurse ensured that each surgeon recruiting to the PROSPECT study completed the questionnaire. Reminders were distributed via email to the lead research nurse.

The responses were manually transferred from a paper questionnaire into a spreadsheet. Each question was presented in a column and the data were coded. Data were analysed in a series of one-way tables. Some sections of the questionnaire were not relevant to every respondent; thus, responses were presented as the proportion of surgeons responding to each question.

## Results

There were 35 centres and 62 surgeons involved in the recruitment of participants to PROSPECT. Of these recruiting surgeons, 90% (*n* = 56 out of 62) completed and returned the questionnaire. The results presented relate to the intraoperative surgical techniques.

### Native tissue anterior and posterior POP repairs

#### Infiltration

Fifty-three surgeons (95%, *n* = 53 out of 56) used infiltration for native tissue anterior and posterior POP repairs. However, there was variation in the volume of fluid infiltrated, ranging from 10 to 80 ml; most surgeons used between 10 and 20 ml (67%, *n* = 35 out of 52).

#### Incision

All but one surgeon performed a midline incision to open the anterior and posterior vaginal walls; this surgeon performed an elliptical incision as routine practice.

#### Dissection

The depth of dissection in the anterior and posterior vaginal walls is summarised in Table 1 and the method used to perform this dissection is shown in Table 2.

**Table 1:** The depth of dissection used for native tissue pelvic organ prolapse (POP) repairs

	Vaginal compartment	
Depth of dissection	Anterior POP repair *n* = 56 (%)	Posterior POP repair *n* = 56 (%)
Dissect fascia of the vaginal epithelium	37	34
Leave fascia on the vaginal epithelium	18	21
Both techniques used	45	45

**Table 2:** The method of fascial dissection used for native tissue POP repairs

Method of fascial dissection	Fascia dissected of the vaginal epithelium *n* = 46 (%)	Fascia left on the vaginal epithelium *n* = 35 (%)
Blunt dissection	4	14
Sharp dissection	63	43
Both methods of dissection	33	43

#### Fascial repair

The most common technique used to repair the fascia for both anterior and posterior POP repairs was midline plication (Table 3). Other techniques included closure of separate fascial defects, paravaginal repair and rectal plication (Table 3). Three surgeons (5%, *n* = 3 out of 56) documented that they performed levator plication as part of their routine native tissue posterior POP repair. When performing a posterior POP repair, 46 surgeons (84%, *n* = 46 out of 55) stated they routinely performed a rectal examination during dissection or at the end of the procedure to ensure sutures did not penetrate the rectal wall.

**Table 3:** The methods of fascial repair used for native tissue POP repairs

Vaginal compartment	Methods of fascial repair			
	Midline plication (%)	Closure of separate defects (%)	Paravaginal repair (%)	Rectal plication (%)
Anterior	46/47 (97)	23/39 (59)	11/44 (25)	NA
Posterior	39/43 (91)	32/43 (74)	NA	17/43 (40)

*NA* not applicable

#### Suturing

The type of suture material used and method of closure of both the skin and fascia are seen in Table 4. All surgeons closed the fascia and the skin separately. An almost equal proportion of surgeons used polydioxanone (PDS; 53%, *n* = 30 out of 56) and polyglactin 910 (Vicryl) sutures (43%, *n =* 24 out of 56) to close the fascia, whereas most surgeons used Vicryl to close the skin (94%, *n* = 53 out of 56). Most surgeons used interrupted sutures on the fascia (66%, *n* = 37 out of 56) and the most frequently used skin closure method was continuous locking sutures (61%, *n* = 34 out of 56).

**Table 4:** The suture material and method of closure of the fascia and skin for native tissue and graft/mesh POP repairs

	Native tissue		Graft/mesh	
	Fascia (%)	Skin (%)	Fascia (%)	Skin (%)
Suture material	(*n* = 56)	(*n* = 56)	(*n* = 34)	(*n* = 45)
PDS	53	2	38	2
Vicryl	43	94	62	94
Both PDS and Vicryl	4	NA	NA	NA
Monocryl	NA	2	NA	2
Polysorb	NA	2	NA	2
Method of closure	(*n* = 56)	(*n* = 56)	(*n* = 40)	(*n* = 51)
Continuous locking	7	61	8	45
Continuous non-locking	25	29	30	35
Interrupted	66	10	60	20
Purse string	2	NA	2	NA

*NA* not applicable

### Graft/mesh POP repairs

Of the 56 surgeons who returned the questionnaire, 3 surgeons did not perform any graft/mesh procedures in this trial. A variety of different grafts/mesh materials were used. All synthetic inserts were type 1 polypropylene from Boston Scientific, Coloplast and Ethicon. Several of the types of mesh inserted were from Ethicon, including Gynemesh, Prosima, and Ultrapro. Seven surgeons did not state the manufacturer of the polypropylene mesh insert. Three manufacturers of biological inserts were used, from Bard, Boston Scientific, and Cook. The mesh kits used were from American Medical Systems (AMS), Bard, Boston Scientific, Cory Bros, and Ethicon. The surgeons’ previous experience of mesh kits usage varied (Table 5).

**Table 5:** Surgeons’ previous mesh kit experience

Number of mesh kits	Surgeons’ previous mesh kit experience, *n* = 50 (%)
<10	34
10–20	18
20–49	30
>50	18

The graft/mesh inserts were soaked in a substance before use by 15 surgeons (30%, *n* = 15 out of 49). These substances included: normal saline (*n* = 11 out of 49), cefotaxime (*n* = 1 out of 49), Savlon (cetrimide and chlorhexidine gluconate; *n* = 1 out of 49), gentamycin (*n* = 1 out of 49) and iodine (*n* = 1 out of 49). The inserts were of a variable size, but the actual measurements were not accurately recorded. Some surgeons described varying the size of the insert depending on the findings during surgery.

The placement of the graft/mesh insert was reported as being inserted below the fascial layer (inlay) by 38 surgeons (79%, *n* = 38 out of 48), above the fascial layer (overlay) by 6 surgeons (13%, *n* = 6 out of 48) and 4 surgeons (8%, *n* = 4 out of 48) performed both techniques. Of those surgeons who only inserted graft/mesh as an overlay, both biological grafts and synthetic mesh materials were used (2 used synthetic mesh only, 2 biological grafts only and 2 both biological and synthetic grafts/mesh).

The graft/mesh inserts were attached either to the pelvic side wall (12%, *n* = 5 out of 43), white line/sacrospinous ligament (86%, *n* = 37 out of 43) or fascia (2%, *n* = 1 out of 43). In addition, 40 surgeons (89%, *n* = 40 out of 45) attached the graft insert to the cervix anteriorly. A branded suture device (Capio) was used by 22 of the recruited surgeons to aid graft/mesh attachment, and the remaining surgeons used another method. As with native tissue repairs, closure of the fascia and skin was performed with a variety of suture types and methods of suturing (Table 4); 7 surgeons closed in one layer only.

### Use of packs

A pack was used following surgery by most surgeons (91%,* n* = 50 out of 55) and it was most commonly soaked in proflavine (acridine-3,6-diamine), an antiseptic lubricant (82%, *n* = 41 out of 50). Other substances used to lubricate the pack included: oestrogen cream (*n* = 3), betadine (povidone-iodine; *n* = 2), dalacin (clindamycin; *n* = 2), hibitane (chlorhexidine; *n* = 4), normal saline (*n* = 1) and Savlon (*n =* 1).

## Discussion

### Summary of findings

To our knowledge, this is the first questionnaire study to report that a significant proportion of surgeons are dissecting beneath the vaginal muscularis (often called fascia by surgeons) in native tissue repairs. It is likely that this “deeper dissection technique” described in this questionnaire survey has developed from techniques used for the insertion of mesh. Typically, mesh is placed below the vaginal muscularis (fascia) directly against the rectum or bladder, as described by Muffly and Barber in 2010 [10]. The traditional plane of dissection is more superficial; the vaginal epithelium is split from the underlying muscularis to enable its plication [7, 11, 12]. Most surgeons in this study continue to use this technique and a past study of Dutch Urogynecologic Society members showed that almost half of the surgeons attempted to “dissect the vaginal mucosa as thin as possible from the bladder” (43%). The remaining Dutch surgeons (47%) considered thickness to be less important and dissected in the “optimal surgical plane” [7]. There was neither a clear definition of this plane nor was the impact of the differing techniques studied.

The volume of infiltration used in this study varied greatly from 10 to 80 ml. The larger volumes of infiltration may again relate to the techniques used for mesh insertion. Larger volumes of infiltration are thought to facilitate a full-thickness dissection of the vaginal muscularis from the bladder or rectum and allow placement of the graft/mesh below it [10]. In older descriptions of native tissue POP repair, no infiltration was used [11, 13]. Infiltration type was not recorded in this study; however, in other studies it has been recorded and the most commonly used infiltrate varied between local anaesthetic [5] and normal saline [7, 14]. Surgeons’ rationale for using local infiltration has been assessed [14], but the reason behind the choice of infiltrate was not evaluated.

In this study, the most commonly performed incision into the vaginal wall was midline; only one surgeon used a different approach, an elliptical incision. In an earlier UK questionnaire study, a much larger range of incisions was reported, including midline, racquet, diamond, inverted T, elliptical and triangular [5]. The reason for the change in practice is unclear.

Most surgeons in this and other studies report repair of the fascia in the midline. This approach was first described by Kelly in 1913 [13] for the management of urinary stress incontinence. Other vaginal fascial repair techniques described include closure of separate fascial defects, paravaginal repair, rectal and levator plication. As in other studies, fewer surgeons performed these methods [5–7]. The reason why midline plication remains the most widely performed method may relate to the success of this method or the ease of repair; however, there is very limited literature assessing how the technique of native tissue repair affects the outcome of surgery [2]. Despite this questionnaire, recommending that levator plication should not be performed, 3 surgeons in this study documented doing this in practice. This technique is associated with an increase in dyspareunia and a decrease in sexual function postoperatively [15]. Unfortunately, we do not know why the 3 surgeons perform the procedure routinely. It may be possible to perform a post hoc analysis of this technique when the PROSPECT trial data are available.

In this study, almost equal proportions of surgeons used Vicryl and PDS for fascial plication, whereas the predominant suture for skin closure was Vicryl. This was different from the techniques described by the Dutch surgeons [7], where Vicryl was the most commonly used suture for fascial plication. Determining why surgeons chose a particular suture material was beyond the scope of the questionnaire. There is very little evidence about the optimal suture material to use. One small RCT of 66 patients [16] found no difference in prolapse symptom scores, but a significantly lower prolapse-related quality of life and urinary incontinence scores when Vicryl was used compared with PDS. A single-centre RCT of pack usage indicated that there was no significant difference in patients’ pain experience postoperatively with or without packing [17]. Future large-scale multicentre RCT studies would help to assess different aspects of technique to provide more evidence on which to base practice.

Also in this study, the most commonly used suture method for fascial plication was interrupted sutures (66%). However, in the Dutch study, there was an equal number of surgeons using interrupted (32%), continuous locking (32%) and continuous sutures (35%) for plication of the fascia [7]. There is no literature demonstrating the best suture method for fascial closure and this may be the reason for the variation seen. A feasibility trial has recently been published that gives some insight into the method of suturing to close the vaginal skin [18]. The pain scores were higher in women 24 hours post-closure when interrupted sutures were used compared with a continuous single suture; however, qualitative research showed that women rated this postoperative pain as insignificant.

Unlike other questionnaire studies [5, 8], this study details the intraoperative techniques used by surgeons for the insertion of graft/mesh. There is a lack of evidence to confirm whether the technique for graft/mesh insertion affects surgical outcome. There is literature that outlines what expert opinion deems to be an acceptable technique [10]. The key practice points described include: catheterisation to drain the bladder; antibiotic usage; avoidance of a T incision when performing concomitant hysterectomy/colporrhaphy; adequate use of infiltration (20–80 ml) to expose the vesicovaginal/rectovaginal space; placement of the graft/mesh below the vaginal muscularis; attachment of the graft/mesh loosely to allow for its contraction; not trimming the vaginal skin; and closure with a non-locking continuous absorbable suture. Given the concern about the integrity of prolapse repairs, it is remarkable that we have so little evidence to define the appropriate management of the key practice points.

Most surgeons in this study placed the graft/mesh below the “fascia” in line with expert opinion. The identification of this space is made possible by a “loss of resistance” technique, where the infiltration is placed in an avascular space between the vaginal muscularis and the bladder/rectum [10]. This creates a fluid bubble that can be identified following dissection through the vaginal wall. A proportion of surgeons in this study (13%) placed graft/mesh above the vaginal muscularis and this included both synthetic and biological grafts/mesh. This variation in technique may be expected to affect the vaginal skin exposure rate, but there is no literature assessing this.

### Study limitations

This surgical technique questionnaire was not fully validated. There were incomplete responses and documentation of multiple methods, which made it difficult to fully evaluate the techniques most commonly used. The layout of the questionnaire may have been a contributing factor to the incomplete responses. In addition, multiple responses may suggest that surgeons might vary their own technique, but from these results we cannot distinguish what determines the approach taken in individual cases. It was beyond the scope of this questionnaire to determine why surgeons chose one technique over another. In addition, we are unable to determine if all surgeons used the same terminology to describe techniques being practiced; for example, the term “fascia” was commonly used, but is poorly defined by surgeons. In view of the variation in surgical techniques between surgeons that has been identified in this study, the pragmatic design of PROSPECT provides the best way of ensuring that results are clinically relevant to UK clinicians at this time.

### Further research

The sample is from 35 centres in the UK, including tertiary teaching hospitals, large district general hospitals and smaller general hospitals. It is therefore probably representative of UK surgeons. However, the survey was conducted to give greater insight into the practices of the surgeons who were contributing to the largest RCT of POP performed to date. Previous surveys of technique have not been associated with outcome data. Very little attention has been paid to surgical technique and how it may influence the outcome of surgery. It was hoped that these data, collected in this specific group, would enable a study of the influence of surgical technique to be performed; however, the variation found in the study and the lack of consistent terminology led us to believe that further qualitative research was required to fully evaluate the variations. A qualitative research study filming surgeons is being undertaken within the PROSPECT trial [9] to create categories of surgical technique to enable a secondary analysis of their influence on outcome.

## Conclusion

In summary, surgical techniques to repair both native tissue and graft/mesh POP repairs varied between UK surgeons. Further research is required to assess whether variation in surgical technique influences the outcome of surgery.

## Appendix 1

### Standardisation of surgical procedures in PROSPECT

Please complete last column to indicate your own practice when performing prolapse surgery (circle or amend). If you vary your technique, please tell us about the one you use most often.

Name………………………………………….

Centre………………………………………….

**1. Native tissue anterior repair**

**Table:**

Date: ……/……/20…..	Procedures	Local practice (variations) *Please circle or amend*
	Midline skin incision through fascial layer and dissection of bladder off cervix/vault	Midline incision
		Other (details)………………….. …………………………………….
	+/− Hydrodissection with 1 in 200,000 adrenaline	Yes/no
		Volume: ……………..ml
Anterior repair Type 1	Dissect fascia off vaginal epithelium	Blunt dissection?
		Sharp dissection?
Anterior repair Type 2	Leave fascia on vaginal skin	Blunt dissection?
	Dissection laterally (but not all the way to the ‘white line’) and sutures placed into fascia in this area	Sharp dissection?
Closure	Fascia and skin closed separately (two-layer closure)	**Fascia** PDS or Vicryl? Fascial sutures: • Continuous locking • Continuous non-locking • Interrupted?
	Plicate fascia in midline if midline defect? Yes/no	
	Separate closure of other fascial defects? Yes/no	
	Paravaginal repair? Yes/no	
	Skin closed	**Skin** PDS or Vicryl? Skin sutures: • Continuous locking • Continuous non-locking • Interrupted?

**2. Native tissue posterior repair**

**Table:**

Date: ……/……/20…..	Procedures	Local practice (variations) *Please circle or amend*
	Midline skin incision through fascial layer	Midline incision
		Other (details)………………….. …………………………………….
	+/− Hydrodissection with 1 in 200,000 adrenaline	Yes/no
		Volume: ……………..ml
Posterior Repair Type 1	Dissect fascia off vaginal epithelium	Blunt dissection?
		Sharp dissection?
Posterior Repair Type 2	Leave fascia on vaginal skin	Blunt dissection?
	Dissection laterally (but not all the way to the sacrospinous ligament) and sutures placed into fascia in this area	Sharp dissection?
Rectal plication	Optional	Yes/no
Closure	Fascia and skin closed separately (two-layer closure)	**Fascia** PDS or Vicryl? Fascial sutures: • Continuous locking • Continuous non-locking • Interrupted?
	Plicate fascia over rectum in midline if midline defect? Yes/no	
	Separate closure of other fascial defects? Yes/no	
	Skin closed	**Skin** PDS or Vicryl? Skin sutures: • Continuous locking • Continuous non-locking • Interrupted?
Levator plication in midline	**Not** to be done as causes dyspareunia	
Rectal examination	Per rectum examination during dissection or after operation to ensure sutures do not penetrate rectal wall	Yes/no

**3. Graft/graft inlay**

**Table:**

Date: ……/……/20…..	Procedures	Local practice (variations) *Please circle or amend*
	Non-absorbable graft	Type: ………………………….
	Biological graft	Type: ………………………….
	Graft kit	Type: ………………………….
	How many kit procedures have you performed?	<10; 10–20; 20–49; > 50
Lateral dissection of pubocervical fascia from vaginal wall	Separate bladder/rectum from fascia using blunt/sharp dissection	Blunt dissection?
		Sharp dissection?
	+/− Hydrodissection with 1 in 200,000 adrenaline	Hydrodissection with 1 in 200,000 adrenaline?
	Dissect fascia off vaginal epithelium	
	[Optional] Dissect out to pelvic side wall (white line or sacrospinous ligament)	Lateral dissection to white line or sacrospinous ligament?
Graft/graft inlay	Cut material to size and lay below fascia (inlay, recommended):	Below fascial layer (**inlay**),
	OR	OR
	Above fascial layer:	Above fascial layer (**overlay**)
	Size of graft/graft:	Size of graft patch:…………cm^2^
	[Optional] soak graft in rifampicin?	Rifampicin?
	OR	OR
	Other fluid?	Other fluid?..........................
	**Attaching the graft**	
	Fix at least 2 PDS/Vicryl sutures or two non-absorbable sutures to pelvic side wall/coccygeus muscle on each side	PDS to attach graft?
		Vicryl to attach graft?
		Non-absorbable suture?
	OR	
	Attach to white line or sacrospinous ligament	Attach to white line (anterior)?
		Attach to sacrospinous ligament (posterior)?
	+/− Capio suturing device	Capio suturing device?
		Yes/no
	(For anterior repair):	Yes/no
	Graft should also be secured to vault or cervix with a suture(s)	
Closure	Two-layer closure (PDS or Vicryl):	**Fascia**
		PDS or Vicryl?
	1. Fascial sutures inserted back from skin edge over graft/graft **(inlay)**	Fascial sutures:
		• Continuous locking
		• Continuous non-locking
		• Interrupted?
	2. Skin closed as second layer **(overlay)**	**Skin**
		PDS or Vicryl?
		Skin sutures:
		• Continuous locking
		• Continuous non-locking
		• Interrupted?

**4. Vaginal packs and lubricants**

**Table:**

Date: ……/……/20…..	Procedures	Local practice (variations) *Please circle or amend*	
	Vaginal pack used for up to 24 h	Yes	No
	(If yes) lubricated?	Oestrogen	Proflavine
		Betadine	Dalacin
		Hibitane	Obstetric cream
		Saline	Savlon
		Aquagel	Dry pack

**5. POP-Q standardisation**

**Table:**

Date: ……/……/20…..	Recommended	Local practice (variations) *Please circle or amend method used most often*
Position	Lithotomy/in leg rests	Lithotomy/in leg rests
		On back on flat bed or table
		On side
		Standing up
		In theatre/under anaesthetic
		Sims speculum
		Plastic speculum (halved)
		Other
Conditions	Bladder status not specified but recorded	Full bladder
		Empty bladder
		Not specified but recorded
		Bladder status not assessed
	Bowel loading recorded	Bowel loading recorded
		Bowel loading not recorded
	Full extent of prolapse seen?	Full extent recorded
		Full extent not recorded
	During Valsalva/pushing down	At rest
		During Valsalva/pushing down
		During cough
	Ruler/measuring stick	Ruler/measuring stick
		Finger measure
		Estimate by eye

## Abbreviations

AMS: American Medical Systems
POP: Pelvic organ prolapse
PROSPECT: PROlapse Surgery: Pragmatic Evaluation and randomized Controlled Trials
POP-Q: Pelvic Organ Prolapse Quantification
PR: Per rectal/rectal examination
RCT: Randomised controlled trial
PDS: Polydioxanone
